# Characteristics of Care and Caregivers of Alzheimer's Patients in Elderly Care Homes: A Qualitative Research

**Published:** 2012-05-30

**Authors:** Sh Yektatalab, M H Kaveh, F Sharif, M Fallahi Khoshknab, P Petramfar

**Affiliations:** 1 Department of Psychiatric Nursing, School of Nursing and Midwifery, Shiraz University of Medical Sciences, Shiraz, Iran; 2 Department of Health Education, Shiraz, Iran; 3 Department of Psychiatric Nursing, School of Nursing and Midwifery, Shiraz University of Medical Sciences, Shiraz, Iran; 4 Department of Nursing, Social Welfare and Rehabilitation University, Tehran, Iran; 5 Department of Neurology, School of Medicine, Shiraz University of Medical Sciences, Shiraz, Iran

**Keywords:** Alzheimer disease, Elderly care home, Perception, Care

## Abstract

**Background:**

Due to the increase in the number of Alzheimer’s patients in Iran and also the limitation of cultural knowledge about caring of these patients, this study was designed to explore the perceptions of Iranian caregivers about caring Alzheimer patients in the elderly care homes.

**Methods:**

A qualitative content analysis method was conducted on two elderly care homes of Shiraz/Iran, during 2009-2011. Fourteen key informants (10 women and 4 men, between 25-35 years of age), who had been working in elderly care homes caring for the elderly with Alzheimer disease for about 1-11 years (Mean=30 months) were selected by purposive sampling method. The caring experience and ability of transferring their experience to others were the main criteria for selection of the participants. They were participated in 2 focus groups and 4 interviews.

**Results:**

Nearly, 800 initial codes were extracted and categorized into 3 groups of "multidimensional care", "going along with the patients" and "need to be professional" and 12 subcategories. Although several aspects of care were mentioned by the participants but the main aspect was physical care. Infantilizing the patients was the main feature of care and caring personality was an important characteristic of caregivers.

**Conclusion:**

An appropriate schedule of care considering main categories and subcategories of this research based on cultural context should be prepared. Moreover, consistent promotion of the schedule, employment of trained staff and plans for continued education for them can improve the quality of care and patient's life in elderly care homes.

## Introduction

Alzheimer disease as one form of dementia is a chronic, progressive and disabling disease which affects cognitive, thought, perception and personality of the patients.[1] In Iran, there are 250000 known cases of Alzheimer disease.[2] Because of the particular feature of the disease, care of these patients is not only a physical procedure, but also a social and psychological phenomenon.[3] Due to the cultural effect, the number of elderly care homes in Iran is limited and usually families take care of their relative at home but today with changes in the nuclear structure of the family and women working outside and also in relation to the level of cognitive impairment and abnormal behavior patterns that change with progression of the disease, the tendency to transfer patients to elderly care homes has increased.[4]

Caregivers have a significant role in providing suitable environment and good care for patients with Alzheimer disease, but evidence showed that few studies have been conducted in nursing paradigm. Algas's research (2007) on Alzheimer disease in nursing paradigm showed that the majority of studies were in other disciplines. Only 3% of these studies were on nursing diagnosis, providing safety, communication and individualized care.[5] Particularly in Iran, to the best of our knowledge, there are no studies on the care of patients with Alzheimer disease. Therefore, the aim of this study was exploring Iranian caregiver's perceptions in elderly care homes which can improve the care and quality of the patient's life.

## Materials and Methods

This qualitative study used the principles of inductive content analysis. We chose qualitative methodology because the purpose was to elucidate the Iranian caregivers' perceptions about providing care to patients with Alzheimer disease.[6] The aim of content analysis was to reach a broad description of the phenomenon, and the consequence of the analysis is concepts or categories that describe the phenomenon.[7] There were five elderly care homes in Shiraz, southwestern Iran. Two elderly care homes which admitted Alzheimer disease patients were purposefully selected; then, 14 key informants (10 women and 4 men, between 25-35 years of age) who had been working in elderly care homes, caring for the elderly with Alzheimer disease for about 1-11 years (Mean=30 months), were selected by purposive sampling method. The caring experience and ability of transferring their experience to others were the main criteria for selection of the participants. Two focus groups and 4 qualitative interviews were conducted. The participants of the focus groups were formal caregivers who had not been educated on nursing or gerontology principles. To complete the data, four other interviews were performed with head nurses and supervisors. Two of them were the holders of bachelor in nursing and two in psychology. Focus group interviews were chosen as they had the advantage of being stimulating, inexpensive and capable of producing rich data.6 The focus groups and interviews started with general questions such as" please explain how you take care of Alzheimer disease patients?" or “please explain your experience from taking care of Alzheimer disease patients" based on the participants' answers, other questions were asked. To probe the informant’s descriptions, more deeply explorative questions were asked such as “give an example, can you explain more, do you mean that?". Each focus group lasted between 90-120 minutes with 5 caregivers and each interview lasted between 60 to 120 minutes. In each session, the researcher guided the discussion, made observation notes about body language, how the group worked together and anything else of interest that could not be detected by tape recording. Saturation of data occurred after two focus groups and four interviews. Two other interviews were done but no new information was gained. The interviews were tape recorded and transcribed verbatim by the interviewer.

For data analysis, inductive content analysis was chosen.[7] In the first step, the transcripts were read repeatedly to gain an overall impression and statements relevant to the goal of the study that were marked, numbered and coded. Then the pool of meaning was revised into categories, named and grouped. Each main category was named using content-characteristic words.[7] The transcripts were checked to ensure that no relevant content was overlooked. The co-authors checked and discussed about the analysis and interpretations until consensus were reached. The quotes were sought in the transcripts to illustrate similarities and differences in the conceptions.

To enhance the credibility and conformability of our qualitative data and analysis, we used maximum variation sampling in sex, age and work experience of participants and member checks. For member checking, after the analysis, a full transcript of the participants' coded interviews with a summary of the emergent theme were given to the participants to ensure about a true representation of their experiences in providing care to Alzheimer patients. Also, prolonged engagement helped data credibility.[8] For further validity check, peer checking was done. Random samples of the interviews were analyzed by three expert supervisors and two independent researchers who were expert in qualitative research. There was an agreement between the different raters. The results were also checked with some of the caregivers who did not participate in the research and they confirmed the transferability of the results as well. The researcher tried to have precise documentation of the direction of research to help other researchers follow the direction of the participants’ experience.

Ethics approval was obtained from the Ethics Committee of Shiraz University of Medical Sciences. Permission for the study was obtained from Shiraz Welfare Organization and managers of elder care homes. Overall and written information was given to the informants and signed.

## Results

About 800 initial codes were gathered from the collection of interviews and categorized into 3 main categories including: “multidimensional care", "going along with the patients" and" need to be professional” and 12 subcategories. Different aspects of care were discussed by the participants. In an analysis performed on this category, six subcategories were discovered as “ physical care”, “psychological care”, “ spiritual care”, “sociocultural care”, “cognitive care” and “familial care” which were discussed.

The findings of this study demonstrated that the content of care focused on daily physical routines in the elder care homes based on the common needs of the elderly residents. Most daily physical cares were devoted to safety, nourishment, toileting and hygiene, protection against harm, administration of medications and rest time. Elderly care home staff members' perceptions regarding this dimension were reflected in the quotation below:

"Dressings were changed in the morning. Food was served at noon and the medication was given after the meal. Then, dinner and medication were given. The tasks were like this. We mostly had controlling and cleaning duties. Taking care of the patients was mainly physical.

"Taking care of the patients was mainly physical and based on daily routines, we did not have much time to talk with the patients”

Mainly, after finishing routine works in the afternoon, the caregivers tried to talk with the patients. For mild Alzheimer disease patients, empathy and sympathy were reported by caregivers but for severe Alzheimer disease patients, because they could not communicate well, this care was to control the patient’s psychological symptoms and make them calm often by unprofessional methods. The patients would stay isolated due to their restrictions of abilities and the staff would communicate more with those who were capable.

“Empathy was very important for the patients. Because they did not live with their family, they need to talk with us but for aggressive and restless patients; we used tranquilizer medication and physical restrain. We could not talk to an aggressive patient”. “The manager asked us about physical care. But during work, we talked to the mild Alzheimer disease patients. We did not know how we could communicate with severe Alzheimer disease patients”

Rituals like praying, attendance in religious places, and connecting to God were organized methods for expressing spirituality in our culture, which were expressed by participants in this study and make them calm”. “We took them to shrines; it was spiritual. They liked memories that were like that”

“Alzheimer disease patients also sat and listened when other patients read prayers. Some of them still remembered prayers”.

“Socio-cultural care was presented in two ways: Group activities inside the institute as parties and group activities in artistic fields, and also entertaining activities outside the institute such as going to parks. This aspect was only used for those patients with mild Alzheimer disease who were controllable outdoors and patients with severe Alzheimer disease were not capable of doing so.

“The elderly, who were more alert and did not need to be taken care of, would go to parks and entertaining places”. “All we did here were to play live music and movies for them once in a while. It was peaceful to hear the sound of light music”.

As for cognitive care for severe Alzheimer disease, the participants pointed out the inefficiency of the intervention. With progression of the disease, cognitive care of the patients became more unprofessional and palliative. Considering the significance of cognitive care in these patients, caregivers had minimum familiarity with this aspect of care.

“Patients with severe Alzheimer disease did not notice anything. For them, there could not be anything done or said. But we went and called him again and asked him how he was. Sometimes he looked. I was not familiar with cognitive care but I asked the patients to talk with me about their past. I thought it helped them to remember their past events”.

“The participants pointed out the importance of care and attention, routine meetings and family’s participation in the care given”. “Some people thought because their patient had Alzheimer disease, it did not make much difference whether to see them or not. But it was shown in their behavior. For instance, Mr. ‘Ch’ felt calm when he saw his family and when they did not visit him he started shouting”.

### Going Along with the Patient

Taking care of patients with Alzheimer disease had some features due to the special characteristics of the disease. This care consists of the features as "To become adapted", "Providing different feelings in the caregivers" and" Infantilizing old adults".

Most of the caregivers in this study stated that at the beginning of their work in the elder care home, they had experienced lack of interest in working with Alzheimer disease patients. By continuing the care, they started to like it and they got to the point of adaptation to their job and discussed and shared their feelings about the patients with their families.

“I have not seen such patients before. It was really difficult in the beginning. I did not even eat food during the first days. It is quite normal now though. We were a family indeed because we slept here at nights and felt at home with them”.

The staff experienced different feelings during their care. Some were negative as feeling of guilt for not providing a fruitful care, sorrow resulting from patient’s death, feeling of sadness for some behaviors of patients and positive feelings such as capability, enjoying the care and satisfaction. Negative feelings were temporary and positive feelings were more highlighted in this study.

“I like taking care from patients with severe and problematic behaviors, because when I helped them, I felt myself capable and important”. “Patients’ repeated requests and sentences made me upset but it was for a short time; I liked my work”.

The participants compared taking care of the patients with that of a baby and they felt better and could tolerate the patients’ behavior more. “Generally, patients with Alzheimer disease were more attractive because their movements were like children. You felt you were taking care of a baby. I saw them as babies. I enjoyed feeding them. I talked with them like a baby. It was not difficult for me at all, as if I was changing a baby’s diapers or I was feeding a baby”.

### Need to be Professional

Three themes emerged in this category consisting of: "level of education and special training","caring personality" and "caregiver's attitude". The participants mentioned that for effective interaction with the patient and providing suitable care, higher education and special training were important. The caregivers in this study had work experience with Alzheimer disease patients but they did not have any specific training, nor had they any academic education in this field. Only two of them (head nurses) were educated in nursing.

“Sometimes I used my personal experience and sometimes I asked the head nurses my questions; one that was educated in nursing. She was more familiar with their psychological and cognitive care”. “With special practical training in the field of gerontology and care, we realized the patients' needs and improve our caring ability”. From participants’ point of view, caring personality was quite influential in caring experience, particularly, characteristics such as empathy, kindness, patience, interest and attention to the patient’s needs.

“If the caregiver did not have patience here, he/she can not even stay for two months because of patients’ yelling and aggression”. Caregiver's attitudes towards the disease had a great influence on the method of care and relationship with the patient. Some of the caregivers had negative attitude towards the symptoms and prognosis of the disease that would affect their communication with the patients. They believed that the patients cannot understand them. As a result, patients would feel more isolated and their symptoms would be intensified. But most of them had a positive attitude toward taking care of these patients due to our cultural context.

“This disease was quite hard and more severe than physical diseases. A mature woman did not know who she was; she did not even know the closest member of her family. It was really hard to commu-nicate with people who did not know anybody”.

“I myself had a good job opportunity, but I liked it here. Their praying had faded many of my problems away”.

## Discussion

Our review of the experiences reported by the participants in this study showed that six aspects of care were mentioned by caregivers but routine daily care was mostly physical. Some earlier studies found that care programs based on ordinary daily routines can help preserve existing skills and slow down the manifestations and progression of dementia.[5][9] However, to provide holistic care for Alzheimer disease patients, other aspects of care such as spiritual, cognitive, psychological, and social elements of the daily programs should be considered. According to Haggestrom et al., many of the caregivers' working hours were spent on physical care, which can detract from efforts to focus on the quality of life.[10] Since in the elderly care homes in this research, this working program was unwritten and based on repetition, suitable care schedules that considered all dimensions of care in elderly care homes should be prepared so that consistent enhancements in the schedule lead to improved quality of care.

The caregivers reported empathy for mild Alzheimer disease patients but they did not have enough knowledge for working with psychological and cognitive problems of severe Alzheimer disease patients; they applied experimental methods like using restraint to keep the patient at ease. Other studies have also mentioned the feeling of discomfort in the patients. Communication and psychological care was considered less in nursing homes.[10][11] Considering the fact that most of the caregivers in this study did not have relevant education and training about Alzheimer disease patients’, this confirms the necessity of selecting expert staff and training courses for them.

In this study, the participants mentioned the importance of spiritual care in Alzheimer disease patients. Facilitating Islamic acts of worship was paramount for religious, older Muslim patients to feel they were well cared for.[12] Also the study of Beusher and Grando (2009) in other contexts, reports that personal faith, prayer, connection to the church, and family support enhanced the ability of people to keep a positive attitude in the early stage of Alzheimer disease. Nonetheless, spirituality had other aspects such as belief system, meaning and purpose of life, connectedness, and transcendence.[13] Therefore, in order to offer a thorough care in spiritual aspect, it is necessary to train caregivers in such aspect.

Socio-cultural care is given to mild Alzheimer disease patients who can be easily taken care of outside; patients with severe Alzheimer disease were not included in this program. In this respect, preparing a suitable and secure condition for all the patients with different levels of Alzheimer disease is necessary to make them feel they were presented in the society and had interactions with others.[14]

In Iranian culture, the family is the most important element and life is usually dominated by family values.[15] This could point out the large number of visitors and self-designated surrogate decision makers for a patient.[16] Iranians feel they need the practical and emotional support that one’s own blood families can provide.[15] The relationship between the members of the family and participating in the care of patients improve their mental status and hence remove the feeling of isolation and depression. Also, the results of Voutilainen et al.'s study demonstrated the need for formulating policies of the ward and training the staff to increase the family involvement in care and to support appropriately.[16]

The studies in other countries emphasized the feeling of burden in the caregivers[17] but in contrast, the participants of this study mentioned the gradual interest in care and working with patients. In Iranian caregivers, helping and caring frail or old people, apart from the social approbation, is considered a labor of love that, in Islam, attracts ample driven reward. This belief as an internal factor is more effective on the continuity of staff work.[18] Also, most of the caregivers reported positive feelings like spiritual growth, capability and positive attitude toward caring frail and old people that can enhance caregivers' interest and dependence on their work. Some participants mentioned temporarily negative feelings such as depression and grief due to patients' problematic behaviors which can be related to their lack of knowledge about Alzheimer disease and strategies to control their behaviors.

Infantilizing old adults is related to child-like behaviors of Alzheimer disease patients and as a mechanism it can facilitate taking care of these patients for caregivers in this study, but according to Thornbury, instead of infantilizing older adults, using a developmental model to facilitate understanding of alleged child-like characteristics observed in Alzheimer disease may be useful in providing insight into the nature of impaired thought processes.[19]

Personal characteristics such as patience and empathy are also discussed in this study. Duffy et al.'s study indicated that dementia patients had especial physical needs. The staff should be patient and recognize the patients' physical and cognitive changes which lead to their inability in their daily activities.[20] Also, Aaron and Gilson believed that empathy with patients mostly depended on the personal characteristics of the personnel.[21] So it is important to select caregiver's base on their appropriate personal characteristics.

The attitude of the caregivers towards the disease has a great influence on care.[22] Caregivers generally believe that in the advanced stages of the disease, patients have less understanding and this attitude could affect the communication with the patients and their care. In collectivist cultures like Iranian culture, relationship and communication is a major factor. So cognitive, psychological and communicational deficit of severe Alzheimer disease patients can affect attitude of caregivers and consequently their needs would not be considered. This attitude is related to caregivers' lack of knowledge about the process of the disease. Norberg et al. in their research referred to staff's negative attitude towards the patients, leading to their job dissatisfaction,[22] but they did not mention in which dimension their participants had negative attitude.

In order to reach to a high quality of care for the Iranian patients with Alzheimer disease, more surveys are recommended to be done in the field of different aspects of care and other core and sub-categories achieved through this study. It is hoped that the results of this research would be taken into account by the authorities so that they provide a holistic care in all dimensions by educated and well trained caregivers and continuous professional development that provide the opportunity to promote the quality of life of patients with Alzheimer disease.
